# Probiotic *Lactobacillus casei Shirota* improves efficacy of amoxicillin-sulbactam against childhood fast breathing pneumonia in a randomized placebo-controlled double blind clinical study

**DOI:** 10.3164/jcbn.17-117

**Published:** 2018-06-08

**Authors:** Bing Li, Junqing Zheng, Xia Zhang, Shan Hong

**Affiliations:** 1 Department of Pediatrics, Jinan Maternity and Child Care Hospital, No.2 Jianguoxiaojingsan Road, Jinan City 250001, Shandong, China; 2 Clinical Laboratory, Jinan Maternity and Child Care Hospital, No.2 Jianguoxiaojingsan Road, Jinan City 250001, Shandong, China; 3 Department of Pediatrics, The Fifth Hospital of Xiamen, No.101 Min’An Road, Xiang’An District, Xiamen City 361101, Fujian, China

**Keywords:** childhood pneumonia, fast breathing pneumonia, amoxicillin-sulbactam, probiotics, *Lactobacillus casei Shirota*

## Abstract

The aim of the present study was to investigate the efficacy of oral administration of probiotic *Lactobacillus casei Shirota* and amoxicillin-sulbactam in treating childhood fast breathing pneumonia. 518 children diagnosed of fast breathing pneumonia were enrolled and randomly assigned to be administered either amoxicillin-sulbactam + *Lactobacillus casei Shirota* or amoxicillin-sulbactam + placebo. Primary outcome was defined as treatment failure before day 3, and secondary outcome was defined as treatment failure during follow-ups on day 6 and 12. Serum levels of tumor necrosis factor-α and interferon-γ were also examined at the end of day 3. Treatment failure rate before day 3 was significantly reduced in amoxicillin-sulbactam + *Lactobacillus casei Shirota* group compared to amoxicillin-sulbactam + placebo group. Serum levels of tumor necrosis factor-α and interferon-γ were both significantly reduced in amoxicillin-sulbactam + placebo group on day 3. On day 6 and 12, although treatment failure rates were higher than on day 3 in both groups, it was still significantly reduced in amoxicillin-sulbactam + *Lactobacillus casei Shirota* group. No severe adverse effects were observed in either treatment group. In conclusion, Probiotic *Lactobacillus casei Shirota*, in combination with amoxicillin-sulbactam, is more effective in treating childhood fast breathing pneumonia, which supports the potential clinical application of *Lactobacillus casei Shirota* as a safe supplement to amoxicillin-sulbactam therapy against childhood fast breathing pneumonia.

## Introduction

Pneumonia is a deteriorating complication that systemically affects multiple organs.^(1)^ It is defined by the World Health Organization (WHO) as a sum of clinical symptoms ranging from fast breathing to more dangerous signs. Since the lung of a child is more susceptible to infections than that of an adult, childhood pneumonia is regarded as a leading cause of childhood morbidity and mortality around the world, particularly in under-developed and developing countries.^(2)^ It has been estimated that approximately 150,000–500,000 infants under one year old die of pneumonia each year.^(3)^

Prescription of antibiotics is recommended by WHO for most childhood pneumonia-like symptoms. In particular, amoxicillin-sulbactam (AS), a novel combination of aminopenicillinbetalactamase inhibitors, has been reported to produce satisfactory therapeutic outcomes against pneumonia in children.^(4)^ However, prescriptions with antibiotics to treat childhood pneumonia can become expensive. For instance, in Pakistan, the average outpatient treatment cost per child per episode of childhood pneumonia was 13 US dollars in 2006, equal to 82% of health expense per person in the same year.^(5)^ Furthermore, in South Asia and sub Saharan Africa, providing all children with antibiotic treatment against pneumonia is estimated to cost approximately 200 million US dollars.^(6)^ Hence, despite recently decreased childhood mortality from pneumonia,^(7)^ pneumonia still remains as a heavy burden and distressing challenge to the health system in under-developed and developing countries.^(8,9)^

Probiotics refer to live microbial ingredients in food that usually exert beneficial effects on human health.^(10,11)^ Consumption of probiotics, often in drinks or capsules as dietary supplements, is safe for healthy individuals as well as patients with various diseases,^(12–14)^ including children who were critically ill.^(15)^ Probiotics have also been reported to exhibit beneficial effects in clinical treatment against pneumonia. For example, Bo *et al.* presented evidences suggesting that use of several probiotics was associated with reduced incidence of ventilator-associated pneumonia (VAP).^(16)^ Similarly, in a randomized controlled multicenter trial among critically ill patients, therapies using the probiotic bacteria *B. Subtilis* and *E. faecalis* were found to be both effective and safe against VAP.^(17)^ A recent pilot trial also supported the use of probiotics in preventing against severe pneumonia.^(18)^ Of particular interest to the current study, in a recent open-label randomized controlled clinical trial conducted among critically ill children on mechanical ventilation, administration of probiotics, such as *Lactobacillus casei Shirota* (LcS), was also shown to reduce incidence of VAP, without complications.^(15)^

Provided with the reported beneficial effects of probiotics against pneumonia, we aimed to examine the clinical efficacy of probiotic LcS to improve the treatment outcome of AS against childhood fast breathing pneumonia in a randomized placebo-controlled double blind study.

## Materials and Methods

### Ethical statements

The current study was designed in accordance with Declaration of Helsinki guidelines, and approved by the Ethical Committee of Jinan Maternity And Child Care Hospital. Signed informed consent forms were obtained from all enrolled patients and/or their family members.

### Patients

During Dec. 2012 and Nov. 2015, a total of 518 children (2–48 months old) diagnosed of fast breathing pneumonia in Jinan Maternity And Child Care Hospital participated in the current study. Diagnosis of fast breathing pneumonia were made by quanlified paediatricians with inclusion criteria as follows: 1) presence of pulmonary infiltrate/consolidation on the frontal and lateral views of chest radiograph; 2) absence of signs of severe or very severe pneumonia 3) respiratory rate equal or higher than 50 breaths/min in children aged 2–11 months and equal or higher than 40 breaths/min in children aged over 12 months.

### Exclusion criteria

All 518 patients initially enrolled were further evaluated by the exclusion criteria as follows: 1) danger signs, including inability to drink, seizure, somnolence, central cyanosis, grunting in a calm child and nasal flaring; 2) lower-chest wall indrawing; 3) malnutrition; 4) chronic debilitating diseases, such as chronic pulmonary illness besides asthma, anatomic abnormalities of the respiratory tract, cancer, progressing neurological disorders, immunological defects, heart disease with clinical repercussion, psychomotor retardation, haemoglobinopathy and liver or kidney disease; 5) other concurrent infections; 6) amoxicillin allergy; 7) history of aspiration; 8) hospitalization during the previous 7 days; 9) any dietary and/or medicinal probiotic supplement during the previous 6 months; 10) amoxicillin or similar antibiotic use during the last 48 h. 64 patients were excluded from the study based on the above criteria.

### Randomization and treatments

The remaining 454 patients after exclusion were assigned, in a random manner with a permuted-block design stratified to their body weight, into two groups (227 in each group): 1) AS + LcS group, who received intravenous administration (i.v.) of AS at a dose of 100 mg kg/day (up to 3 g/day) every 8 h (Q8H), plus oral administration of skimmed milk containing a minimum of 6 × 10^9^ colony-forming units (CFU) of LcS; 2) AS + placebo group, who received i.v. AS at a dose of 100 mg kg/day (up to 3 g/day) Q8H, plus skimmed milk as placebo. All patients were treated on a daily basis for 3 consecutive days. Drugs including both types of skimmed milk were prepared by investigators blind to the notified not to consume any food supplement or medication containing probiotics, except those supplied by the investigators throughout the study. 12 patients from the AS + LcS group and 14 patients from the AS + placebo group did not comply to such instruction and were thus dropped out from the study. Their data were not included from the final analysis.

### Serum cytokine measurements

Blood samples (2–5 ml) were collected from all patients at the end of day 3 into test tubes with 0.1% EDTA, and then immediately centrifuged and stored at −80°C until further use. Serum concentrations of interferon-γ (IFN-γ) and tumor necrosis factor α (TNF-α) were assessed with commercial assay kit from Harbin Pharmaceutical Group (Harbin, China) following manufacturer’s instructions.

### Treatment outcomes

The primary outcome was defined as treatment failure up to the third day, with patients displaying any of the following symptoms: 1) persistent axillary temperature >37.5°C; 2) persistence of tachypnea, with respiratory rate ≥50 breaths/min in children aged 2–11 months and respiratory rate ≥40 breaths/min in children aged ≥12 months; 3) development of danger signs, including chest indrawing, inability to drink, grunting, cyanosis, nasal flaring, seizure and somnolence; 4) development of serious adverse reactions. Secondary outcome was defined as treatment failure at day 6 and 12 upon follow up, with patients displaying all of the above complications, pluspersistent cough and recurrent fever.

### Statistical analysis

Sample size of treatment groups was estimated using established statistical analysis method.^(19)^ A sample size of 454 patients, 227 in each arm, is sufficient to detect a clinically important difference of 24% between two treatment groups in terms of primary outcome using a two-tailed z-test of proportions between two groups with 80% power and a significance of 0.05. This 24% difference represents an 80% reaching primary outcome in AS + LcS group and 56% reaching primary outcome in AS + placebo group. Two tailed Students *t* test was utilized to determine statistical differences between the two treatments, and *p* value less than 0.05 was considered statistically significant. Data analysis was performed using SPSS 18.0 software package (SPSS Inc., Chicago, IL).

## Results

**Fig. 1:**
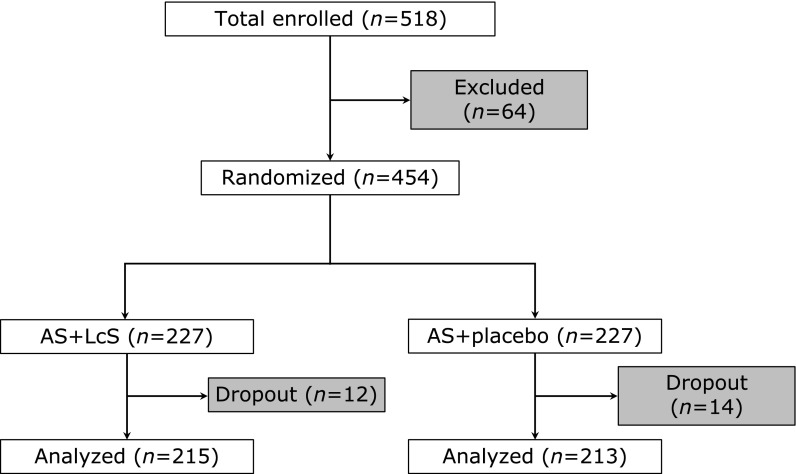
Study design.

Design of the current study was illustrated in Fig. 1. A total of 518 children (2–48 months old) were diagnosed of fast breathing pneumonia, including 64 patients who were later excluded based on the exclusion criteria. The remaining 454 patients were subsequently assigned into two treatment groups in a random fashion: 227 patients in the AS + LcS group and 227 patients in the AS + placebo group. 12 and 14 patients from AS + LcS group and AS + placebo group, respectively, were dropped out from the study before end of study.

Comparison of characteristics of all eligible patients from the two treatment groups was shown in Table 1. No significant differences were obsereved with regard to age, gender or symptoms at enrollment including tachypnea, fever, reduced pulmonary expansion, chest retraction, rhonchi, wheezing and crackles.

Treatment failure up to day 3 was defined as the primary outcome, and the specific causes for failures were listed in Table 2. There was a significantly lower number of patients in the AS + LcS group manifesting fever, chest indrawing and tachypnea than those in the AS + placebo group. No serious adverse reaction to either treatment was observed.

**Fig. 2:**
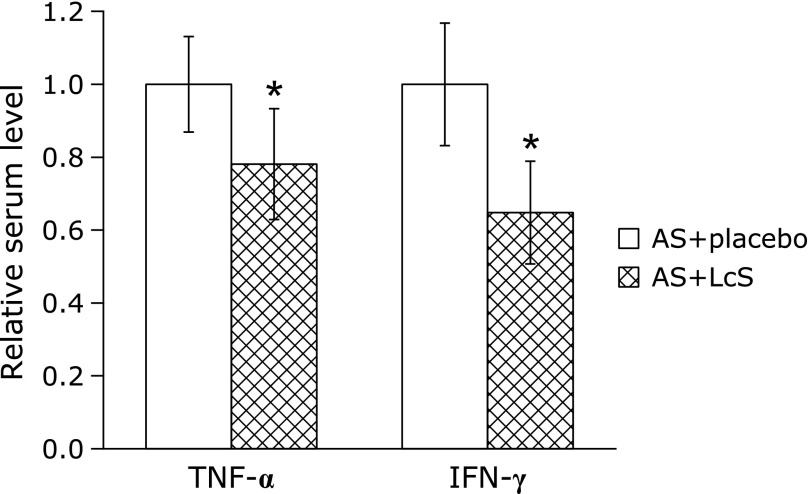
Serum levels of TNF-α and IFN-γ were analyzed patients of the two treatment groups. Values are mean ± SD, *p<0.05 compared with AS + placebo group, respectively.

As pro-inflammatory cytokines TNF-α in IFN-γ have been reported to be involved in childhood severe and non-severe community acquired pneumonia,^(20)^ we were curious whether these two cytokines were potentially implicated in childhood fast breathing pneumonia. In this context, at the end of treatment day 3, blood samples were collected to analyze serum levels of TNF-α and IFN-γ. As shown in Fig. 2, both TNF-α and IFN-γ levels in the serum were significantly reduced in AS + LcS group patients compared to those in AS + placebo group.

Patients were followed by clinic revisits on day 6 and 12 after the start of study, and treatment failures on both revisits were defined as secondary outcome (Table 3). Number of patients in the AS + LcS group manifesting recurrent fever and persistent cough on day 6 was significantly lower than those in the AS + placebo group. In addition, although the number of treatment failure increased in patients from both treatments on day 12, the incidence of treatment failures in the AS + LcS group was nevertheless significantly lower than that in the AS + placebo group.

## Discussion

Pneumonia is a serious disorder systemically affecting a variety of organs. Several risk factors have been implicated in childhood pneumonia, such as lack of exclusive breastfeeding, cigarette use and in particular exposure to air pollution in rapidly developing economies.^(21,22)^ A recent investigation revealed that exposure to particulate matter such as PM2.5 was associated with hospitalizations for respiratory diseases,^(23)^ indicating air pollutants contribute to multiple respiratory diseases including pneumonia.^(24–27)^ With the recent vast economic growth in China, air pollution, for instance from vehiclle exhaust, is thought to contribute to the increased incidence rate of pneumonia.^(28,29)^ Hence, it is urgent to improve the current clinical management of pneumonia, particularly among children, because lungs of children appear more susceptible to air pollution and/or infections than adults, and even air pollution from cooking with solid fuels was reportedly associated with pre-mature deaths from pneumonia for children under 5 years old.^(30)^

In the current clinical study, we focused on the milder form of childhood pneumonia termed childhood fast breathing pneumonia, for which WHO recommended oral amoxicillin as a priority treatment.^(31)^ Unfortunately, based on recent clincial reports among children with fast breathing pneumonia, the efficacy of oral amoxicillin was still far from satisfactory.^(32)^ On the other hand, the wide anti-microbial proporty of AS makes it an effective therapy against childhood pneumonia.^(4,33)^ However, its clinical efficacy against childhood fast breathing pneumonia has not been investigated, and a safer and more effective agent to supplement AS is also needed. To this end, we examined the probiotic LcS, that has been widely used as a dietary supplement and reportedly exerts beneficial effects in various models of respiratory disorders, including pneumonia.^(15)^ Our results suggested that i.v. AS supplemented with oral LcS greatly improved the treatment outcome against childhood fast breathing pneumonia, as revealed by the greatly decreased treatment failure incidence up to 12 days following the combinational therapy. Addtionally, as a common and commercially available probiotic, LcS is intrisincally safe. In fact, a number of clinical trials have proven the clinical safety of LcS.^(12–15)^ Agreeing with those reports, no adverse reaction was observed in our current study.

With data from our current study, question has left to be answered regarding the molecular mechanism responsible for the synergistic action of LcS with AS against childhood fast breathing pneumonia. LcS has been demonstrated to possess potent anti-inflammatory properties in various disease models. For instance, LcS was shown to attenuate inflammatory joint damage in collagen-induced arthritis,^(34)^ as well as to reduce pain, inflammation and degradation of articular cartilage.^(35)^ In a streptozotocin-induced diabetic rat model, LcS significantly lowered blood levels of pro-inflammatory cytokines such as interleukin-6 and C-reactive protein.^(36)^ Importantly, in an allergy mouse model, mice fed with LcS showed attenuated lung inflammation and reduced levels of pro-inflammatory cytokines, including TNF-α and IFN-γ, in bronchoalveolar lavage fluid.^(37)^ This report is consistent with results of our current study, where both TNF-α and IFN-γ levels in the serum were significantly reduced in AS + LcS group patients compared to AS + placebo group patients, indicating inhibited inflammation. Based on previous reports, as well as our own data, regarding both the clinical efficacy and underlying mechanisms of LcS, we speculate that LcS could also exert synergistic action with AS in the treatment of childhood fast breathing pneumonia through its anti-inflammatory properties.

In conclusion, in the current randomized placebo-controlled double blind clinical study, we presented results regarding the efficacy of LcS to supplement AS in the clinical treatment of childhood fast breathing pneumonia. Supplementing LcS to AS prescription suppressed inflammation and greatly reduced incidences of treatment failure compared to AS alone. To our best knowledge, our study is the first report providing clinical data to support the role of LcS as a potent adjuvent of AS therapy in the management of childhood pneumonia without apparent adverse effects.

## Figures and Tables

**Table 1:** Comparison of baseline characteristics between analyzed patients of the two treatment groups

	AS + LcS (*n* = 215)	AS + placebo (*n* = 213)
Gender (male/female)	104/111	107/106
Median age, range (months)	27.3 (2–48)	26.8 (2–48)
Symptoms at enrollment		
Fever	67	71
Tachypnea	128	133
Chest retraction	12	10
Reduced pulmonary expansion	47	51
Rhonchi	154	149
Wheezing	86	91
Crackles	124	112

**Table 2:** Primary outcome: comparison of treatment failure up to day 3 between analyzed patients of the two treatment groups

	AS + LcS (*n* = 215)	AS + placebo (*n* = 213)	*p*
Fever	10	22	<0.05
Tachypnea	19	54	<0.05
Chest indrawing	4	9	<0.05
Adverse drug reaction	0	0	N.A.
Total number reached primary outcome	182	128	<0.05

N.A. not applicable.

**Table 3:** Secondary outcome: comparison of treatment failure at day 6 and 12 between analyzed patients of the two treatment groups

	Day 6				Day 12		
	AS + LcS (*n* = 182)	AS + placebo (*n* = 128)	*p*		AS + LcS (*n*=182)	AS + placebo (*n*=128)	*p*
Persistent cough	11	26	<0.05		19	41	<0.05
Recurrent fever	9	19	<0.05		18	39	<0.05
Total number reached secondary outcome	162	83	<0.05		145	48	<0.05

## References

[1] Hooven TA, Polin RA (2017). Pneumonia. Semin Fetal Neonatal Med.

[2] Boloursaz MR, Lotfian F, Aghahosseini F (2013). Epidemiology of lower respiratory tract infections in children. J Compr Ped.

[3] GBD 2015 Mortality and Causes of Death Collaborators. (2016). Global, regional, and national life expectancy, all-cause mortality, and cause-specific mortality for 249 causes of death, 1980–2015: a systematic analysis for the Global Burden of Disease Study 2015. Lancet.

[4] Lovera D, Arbo A (2005). Treatment of childhood complicated community-acquired pneumonia with amoxicillin/sulbactam. J Chemother.

[5] Hussain H, Waters H, Omer SB (2006). The cost of treatment for child pneumonias and meningitis in the Northern Areas of Pakistan. Int J Health Plann Manage.

[6] Edejer TT, Aikins M, Black R, Wolfson L, Hutubessy R, Evans DB (2005). Cost effectiveness analysis of strategies for child health in developing countries. BMJ.

[7] GBD 2013 Mortality and Causes of Death Collaborators. (2015). Global, regional, and national age-sex specific all-cause and cause-specific mortality for 240 causes of death, 1990–2013: a systematic analysis for the Global Burden of Disease Study 2013. Lancet.

[8] Lee KS, Park SC, Khoshnood B, Hsieh HL, Mittendorf R (1997). Human development index as a predictor of infant and maternal mortality rates. J Pediatr.

[9] Duke T (2005). Neonatal pneumonia in developing countries. Arch Dis Child Fetal Neonatal Ed.

[10] Fuller R (1989). Probiotics in man and animals. J Appl Bacteriol.

[11] Salminen S, Ouwehand A, Benno Y, Lee YK (1999). Probiotics: how should they be defined?. Trends Food Sci Tech.

[12] Matsuzaki T, Saito M, Usuku K (2005). A prospective uncontrolled trial of fermented milk drink containing viable *Lactobacillus casei* strain Shirota in the treatment of HTLV-1 associated myelopathy/tropical spastic paraparesis. J Neurol Sci.

[13] Stadlbauer V, Mookerjee RP, Hodges S, Wright GA, Davies NA, Jalan R (2008). Effect of probiotic treatment on deranged neutrophil function and cytokine responses in patients with compensated alcoholic cirrhosis. J Hepatol.

[14] Dong H, Rowland I, Thomas LV, Yaqoob P (2013). Immunomodulatory effects of a probiotic drink containing Lactobacillus casei Shirota in healthy older volunteers. Eur J Nutr.

[15] Banupriya B, Biswal N, Srinivasaraghavan R, Narayanan P, Mandal J (2015). Probiotic prophylaxis to prevent ventilator associated pneumonia (VAP) in children on mechanical ventilation: an open-label randomized controlled trial. Intensive Care Med.

[16] Bo L, Li J, Tao T (2014). Probiotics for preventing ventilator-associated pneumonia. Cochrane Database Syst Rev.

[17] Zeng J, Wang CT, Zhang FS (2016). Effect of probiotics on the incidence of ventilator-associated pneumonia in critically ill patients: a randomized controlled multicenter trial. Intensive Care Med.

[18] Cook DJ, Johnstone J, Marshall JC (2016). Probiotics: Prevention of Severe Pneumonia and Endotracheal Colonization Trial-PROSPECT: a pilot trial. Trials.

[19] Sakpal TV (2010). Sample size estimation in clinical trial. Perspect Clin Res.

[20] Haugen J, Chandyo RK, Brokstad KA (2015). Cytokine concentrations in plasma from children with severe and non-severe community acquired pneumonia. PLoS One.

[21] PrayGod G, Mukerebe C, Magawa R, Jeremiah K, Török ME (2016). Indoor air pollution and delayed measles vaccination increase the risk of severe pneumonia in children: results from a case-control study in Mwanza, Tanzania. PLoS One.

[22] Nguyen TK, Tran TH, Roberts CL, Fox GJ, Graham SM, Marais BJ (2017). Risk factors for child pneumonia - focus on the Western Pacific Region. Paediatr Respir Rev.

[23] Nascimento LF, Vieira LC, Mantovani KC, Moreira DS (2016). Air pollution and respiratory diseases: ecological time series. Sao Paulo Med J.

[24] Nordenhall C, Pourazar J, Blomberg A, Levin JO, Sandstrom T, Adelroth E (2000). Airway inflammation following exposure to diesel exhaust: a study of time kinetics using induced sputum. Eur Respir J.

[25] Bosson J, Pourazar J, Forsberg B, Adelroth E, Sandstrom T, Blomberg A (2007). Ozone enhances the airway inflammation initiated by diesel exhaust. Respir Med.

[26] Patto NV, Nascimento LF, Mantovani KC, Vieira LC, Moreira DS (2016). Exposure to fine particulate matter and hospital admissions due to pneumonia: Effects on the number of hospital admissions and its costs. Rev Assoc Med Bras (1992)..

[27] Vodonos A, Kloog I, Boehm L, Novack V (2016). The impact of exposure to particulate air pollution from non-anthropogenic sources on hospital admissions due to pneumonia. Eur Respir J.

[28] Coffin DL, Blommer EJ (1967). Acute toxicity of irradiated auto exhaust. Its indication by enhancement of mortality from streptococcal pneumonia. Arch Environ Health.

[29] Deng W, Hu Q, Liu T (2017). Primary particulate emissions and secondary organic aerosol (SOA) formation from idling diesel vehicle exhaust in China. Sci Total Environ.

[30] Mortimer K, Ndamala CB, Naunje AW (2017). A cleaner burning biomass-fuelled cookstove intervention to prevent pneumonia in children under 5 years old in rural Malawi (the Cooking and Pneumonia Study): a cluster randomised controlled trial. Lancet.

[31] World Health Organization. (2012). Recommendations for Management of Common Childhood Conditions: Evidence for Technical Update of Pocket Book Recommendations: Newborn Conditions, Dysentery, Pneumonia, Oxygen Use and Delivery, Common Causes of Fever, Severe Acute Malnutrition and Supportive Care. WHO Guidelines Approved by the Guidelines Review Committee.

[32] Vilas-Boas AL, Fontoura MS, Xavier-Souza G, et al.; PNEUMOPAC-Efficacy Study Group (2014). Comparison of oral amoxicillin given thrice or twice daily to children between 2 and 59 months old with non-severe pneumonia: a randomized controlled trial. J Antimicrob Chemother.

[33] Aronoff SC, Jacobs MR, Johenning S, Yamabe S (1984). Comparative activities of the beta-lactamase inhibitors YTR 830, sodium clavulanate, and sulbactam combined with amoxicillin or ampicillin. Antimicrob Agents Chemother.

[34] Amdekar S, Singh V, Singh R, Sharma P, Keshav P, Kumar A (2011). *Lactobacillus casei* reduces the inflammatory joint damage associated with collagen-induced arthritis (CIA) by reducing the pro-inflammatory cytokines: *Lactobacillus casei:* COX-2 inhibitor. J Clin Immunol.

[35] So JS, Song MK, Kwon HK (2011). *Lactobacillus casei* enhances type II collagen/glucosamine-mediated suppression of inflammatory responses in experimental osteoarthritis. Life Sci.

[36] Zarfeshani A, Khaza'ai H, Mohd Ali R, Hambali Z, Wahle KW, Mutalib MS (2011). Effect of *Lactobacillus casei* on the production of pro-inflammatory markers in streptozotocin-induced diabetic rats. Probiotics Antimicrob Proteins.

[37] Lim LH, Li HY, Huang CH, Lee BW, Lee YK, Chua KY (2009). The effects of heat-killed wild-type *Lactobacillus casei* Shirota on allergic immune responses in an allergy mouse model. Int Arch Allergy Immunol.

